# Peptidoglycan recognition in Drosophila is mediated by LysMD3/4

**DOI:** 10.1016/j.jbc.2023.104758

**Published:** 2023-04-26

**Authors:** Mark Snee, Jason Wever, Jennifer Guyton, Ryan Beehler-Evans, Christine C. Yokoyama, Craig A. Micchelli

**Affiliations:** 1 Department of Developmental Biology, Washington University School of Medicine, St Louis, Missouri, USA; 2 Department of Pathology and Immunology, Washington University School of Medicine, St Louis, Missouri, USA

**Keywords:** peptidoglycan, pattern recognition receptor, innate immunity, NF-kappa B, lysin motif domain, Drosophila, human

## Abstract

Microbial recognition is a key step in regulating the immune signaling pathways of multicellular organisms. Peptidoglycan, a component of the bacterial cell wall, exhibits immune stimulating activity in both plants and animals. Lysin motif domain (LysMD) family proteins are ancient peptidoglycan receptors that function in bacteriophage and plants. This report focuses on defining the role of LysMD-containing proteins in animals. Here, we characterize a novel transmembrane LysMD family protein. Loss-of-function mutations at the *lysMD3/4* locus in Drosophila are associated with systemic innate immune activation following challenge, so we refer to this gene as immune active (*ima*). We show that Ima selectively binds peptidoglycan, is enriched in cell membranes, and is necessary to regulate terminal innate immune effectors through an NF-kB–dependent pathway. Hence, Ima fulfills the key criteria of a peptidoglycan pattern recognition receptor. The human Ima ortholog, hLysMD3, exhibits similar biochemical properties. Together, these findings establish LysMD3/4 as the founding member of a novel family of animal peptidoglycan recognition proteins.

Peptidoglycan is a bacterial cell wall–derived carbohydrate and one of the most abundant polymers found in nature (1). It surrounds the cytoplasmic membrane forming a mesh-like layer (2, 3). Peptidoglycan is responsible for the rigidity and shape of bacterial cells, so disrupting either its biogenesis (*e.g.* antibiotics) or integrity (*e.g.* lysozyme) results in bacterial cell lysis. Chemically, peptidoglycan is a polymer consisting of glycan strands cross-linked *via* short peptide stems. The unmodified, polymerized glycan strand consists of alternating units of β-(1,4) linked GlcNAc and *N*-acetylmuramic acid residues. The peptide stem consists of 2 to 5 amino acid residues, which can become cross-linked to the peptide stem of an adjacent glycan strand, either directly or *via* an interpeptide bridge (4). Variations in the composition of both the glycan strands and the peptide bridge occur between bacterial species and growth conditions (5, 6). While peptidoglycan is produced in prokaryotes, it is not synthesized by metazoans, thus providing a unique chemical signature indicating bacterial presence.

The lysin motif domain (LysMD) was first identified as a 44 amino acid direct repeat in the bacteriophage φ29 gene 15, which encodes a lysozyme (7). Similar sequences were subsequently isolated from *Streptococcus faecalis* in a screen to identify autolysins involved in bacterial cell wall turnover (8). The authors noted the sequence similarity to the φ29 gene 15 lysozyme repeats previously described by Garvey *et al.* (7), and on this basis, they first suggested that the region could be responsible for mediating peptidoglycan binding. The structure of the LysM domain was solved for an *Escherichia coli* gene encoding a transglycosylase, which indicated the presence of a βααβ secondary structure with the two α helices packing onto the same side of an antiparallel β sheet (9). A potential ligand-binding site was also described on the protein surface. The possibility that LysMD-containing proteins could bind peptidoglycan was directly tested in Lactococcus *lactis* where three LysMD repeats in the AcmA autolysin enzyme were found to be necessary for peptidoglycan binding (10). Subsequent sequence analysis has revealed the widespread distribution of the LysMD in many organisms, with the notable exception of archaea (11, 12).

In addition to the previously defined roles in prokaryotes, LysMD-containing proteins have also emerged as one of the two main receptor types mediating the immune response in plants (13, 14). Plant LysMD-containing proteins have been shown to directly bind peptidoglycan and regulate different aspects of immune signaling (15, 16). However, since *bona fide* peptidoglycan-binding proteins identified in animals do not contain LysM domains (17, 18, 19), peptidoglycan recognition is thought to have evolved through convergent evolution (15). Here, we directly test the role of LysMD-containing proteins in animals. We demonstrate that a transmembrane protein containing a single LysM domain binds to peptidoglycan, is enriched in cell membranes, and functions to regulate the expression of terminal innate immune effectors in a pathogen challenge model. We conclude that LysMD-mediated peptidoglycan recognition is a conserved mechanism of immune regulation in animals.

## Results

### Ima binds peptidoglycan *in vitro*

**Figure 1:**
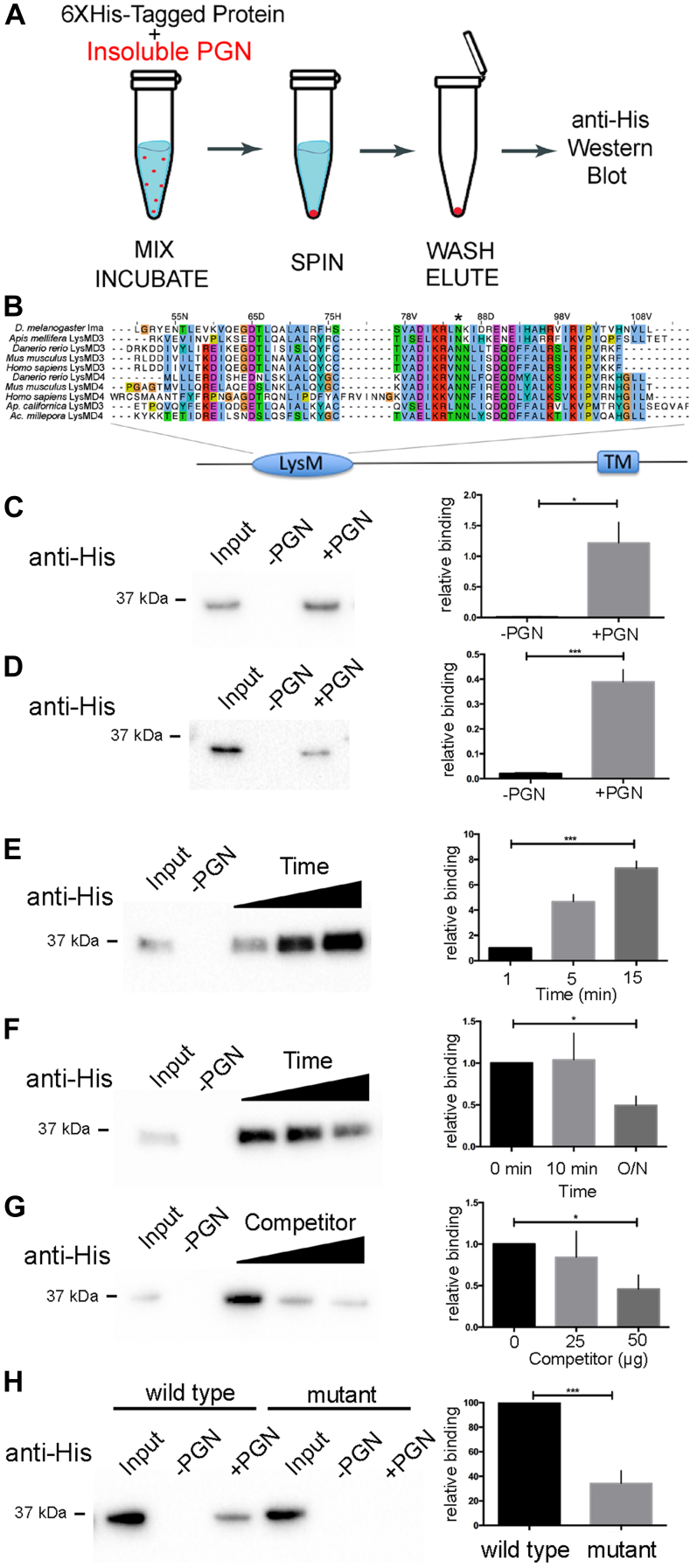
**LysMD proteins bind gram-negative peptidoglycan**. *A*, an affinity-binding assay was used to measure the interaction between recombinant LysMD-containing proteins and insoluble Gram-negative peptidoglycan (PGN). *B*, sequence comparison of LysM domains from different species showing regions of amino acid conservation. *C*–*H*, Western blots were probed with anti-His antibodies to detect 6XHis-tagged proteins and signal intensity was quantified. Two percent of the total 6XHis-tagged protein present in each binding reaction was directly loaded on the gel alone, as a positive control for detection of 6XHis-tagged protein (Input). *C*, 6XHis-Ima was incubated in the presence or absence of PGN (±PGN); n = 3 independent trials. Values normalized to input. *D*, 6XHis-humanLysMD3 was incubated in the presence or absence of PGN (±PGN); n = 5 independent trials. Values normalized to input. *E*, 6XHis-Ima was incubated in the presence or absence of PGN (±PGN) for different periods of time. The effect of varying incubation time on the extent of PGN binding was measured; n = 3 independent trials. Values normalized to binding at 1 min. *F*, 6XHis-Ima was incubated in the presence or absence of PGN (±PGN). The effect of increasing wash time prior to elution on the extent of PGN binding was measured; n = 3 independent trials. Values normalized to binding at 0 min dissociation. *G*, 6XHis-Ima was incubated in the presence or absence of PGN (±PGN). The effect of increasing soluble competitor on PGN binding was measured; n = 4 independent trials. Values normalized to 0 μg competitor. *H*, 6XHis-Ima and 6XHis-Ima[N85A] point mutant (*asterisk* in *B*) were incubated in the presence or absence of PGN (±PGN). The effect of mutating a single conserved residue in the LysM domain on PGN binding was measured; n = 6 independent trials. Values normalized to WT binding. ∗*p* < 0.05, ∗∗∗*p* < 0.001, unpaired Students *t* test; Error bars, SE. ima, immune active; LysMD, lysin motif domain.

The Drosophila *lysMD3/4* locus, immune active (*ima*), is predicted to encode a transmembrane protein containing a single LysM domain with homology to LysMD family proteins found in other organisms (Fig. S1*A*). Sequence analysis indicates that the ancestral *lysMD3/4* gene underwent duplication in vertebrates, giving rise to the *lysMD3* and *lysMD4* genes of the lineage and that *lysMD3* is the closest *ima* ortholog in the human genome (Fig. S1, *B* and *C*). To evaluate the function of these gene products, an *in vitro* affinity-binding assay was used to measure the extent to which animal LysMD-containing proteins could interact with peptidoglycan (20) (Fig. 1*A*). Both Ima and hLysMD3 were tested in the assay. We screened recombinant 6XHis-tagged proteins for binding to insoluble peptidoglycan and analyzed the pellet using SDS-PAGE and immunoblotting (Figs. 1*A* and S2, *A* and *B*). These experiments reveal that 6XHis-Ima binds insoluble peptidoglycan derived from gram-negative bacteria *in vitro*, but not from gram-positive bacteria (Figs. 1*C* and S2, *C* and *D*). Quantification showed that the extent of peptidoglycan binding is significant compared to controls (Figs. 1*C* and S2, *C* and *D*). Similar results were obtained for 6XHis-hLysMD3 (Figs. 1*D* and S2, *C* and *D*). Importantly, binding of 6XHis-Ima to peptidoglycan was both rapid and reversible (Figs. 1, *E* and *F* and S2, *E* and *F*). Furthermore, binding of 6XHis-Ima to insoluble peptidoglycan was attenuated in a dose-dependent manner through the addition of soluble competitor (Fig. 1*G*). We next asked if the LysM domain of Ima itself is required for peptidoglycan binding. Site-directed mutagenesis was first used to mutate a single conserved asparagine residue to alanine, within the LysM domain (Fig. 1*B*, asterisk). The affinity-binding assay was then used to determine the extent to which the 6XHis-Ima^ΔN85A^ mutant protein binds peptidoglycan. In contrast to 6XHis-Ima controls, 6XHis-Ima^ΔN85A^ mutants displayed a significant decrease in peptidoglycan binding (Fig. 1*H*). Taken together, these results demonstrate that Ima protein binds peptidoglycan derived from gram-negative bacteria in an LysMD-dependent manner.

### Ima is localized to cell membranes

**Figure 2:**
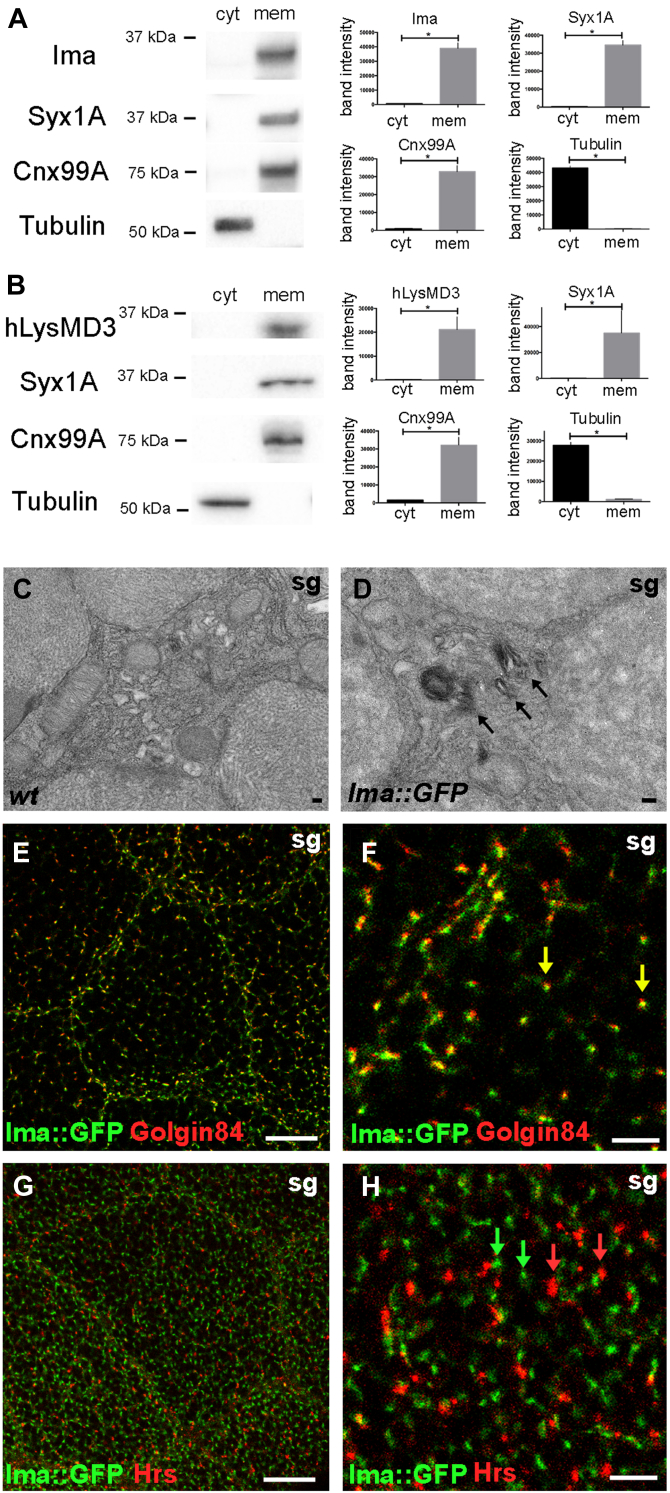
**LysMD proteins localize to cell membranes.***A* and *B*, biochemical separation of whole cell lysates from adult flies into cytoplasmic (cyt) and membrane (mem) subfractions. Fractions were analyzed for protein components using Western blotting; Syx1A, plasma membrane marker; Cnx99A, ER membrane marker; Tubulin, cytoplasm marker. Mann-Whitney, ∗*p* < 0.05. Band intensity is plotted in arbitrary fluorescence units. *A*, WT flies; anti-Ima; n = 4 independent trials. *B*, the human *ima* homolog, *hlysMD3*, was expressed in WT *Drosophila* using the ubiquitous *da-gal4* promoter driving the *UAS-hlysMD3* transgene; anti-hLysMD3; n = 4 independent trials. *C* and *D*, distribution of Ima-GFP using immuno-EM. *Arrows* show stacked Golgi membranes in larval salivary gland (sg) cells. The scale bar represents 100 nm. *E*–*H*, colocalization of Ima-GFP with endomembrane markers using immunohistocytochemistry and confocal microscopy in larval salivary gland (sg) cells. Golgin-84, marker of Golgi rims; Hrs, early endosome. *E* and *G*, the scale bar represents 20 μm. *F* and *H*, the scale bar represents 5 μm. ER, endoplasmic reticulum; ima, immune active; LysMD, Lysin motif domain.

A biochemical fractionation assay was next used to determine the cellular compartment to which the endogenous Ima protein is localized (21, 22). Specific antiserum was first raised against Ima protein (Supporting information, experimental procedures). Next, whole fly lysates were treated with detergent to distinguish membrane from cytoplasmic subfractions, which were then analyzed using Western blotting. Our results show that Ima is detected in the cellular fraction enriched for membrane-localized marker proteins, but not in the cytosolic fraction (Figs. 2*A* and S3*A*). Similar fractionation results were obtained for the hLysMD3 protein, when it was heterologously expressed in Drosophila tissues using a ubiquitous promoter (Figs. 2*B* and S3*B*).

We next generated an in-frame GFP-tagged Ima protein (*ima-GFP*) expressed from the endogenous genomic locus using CRISPR/Cas9 editing technology (23, 24). The localization of Ima-GFP was first analyzed using immunoelectron microscopy, which revealed a strong signal present in the morphologically distinct Golgi apparatus of salivary gland epithelial cells, which are classically used to characterize subcellular protein localization owing to their large size (Fig. 2, *C* and *D*). Moreover, double-labeling immunofluorescence studies confirmed this initial finding and showed Ima-GFP colocalization with the Golgi-associated marker golgin-84, but not with the endosome marker Hrs (25) (Figs. 2, *E*–*H* and S4, *A*–*D*). Taken together, these studies demonstrate that Ima protein is enriched in the cellular membrane fraction and selectively colocalizes with markers of the endomembrane system.

### Ima regulates innate immune signaling

To study the requirement of Ima in the response to bacterial challenge *in vivo*, we used CRISPR/Cas9 genome editing to delete the LysM domain from the previously uncharacterized Drosophila *lysMD3/4* locus and generate the *ima*^*Δ*^ mutation (Fig. S5, *A* and *B*). This resulted in an out-of-frame deletion in the coding region and decreased Ima protein levels (Fig. S5*C*). Flies homozygous for the *ima*^*Δ*^ mutation deletion were viable and appeared morphologically normal under standard laboratory culture conditions.

**Figure 3:**
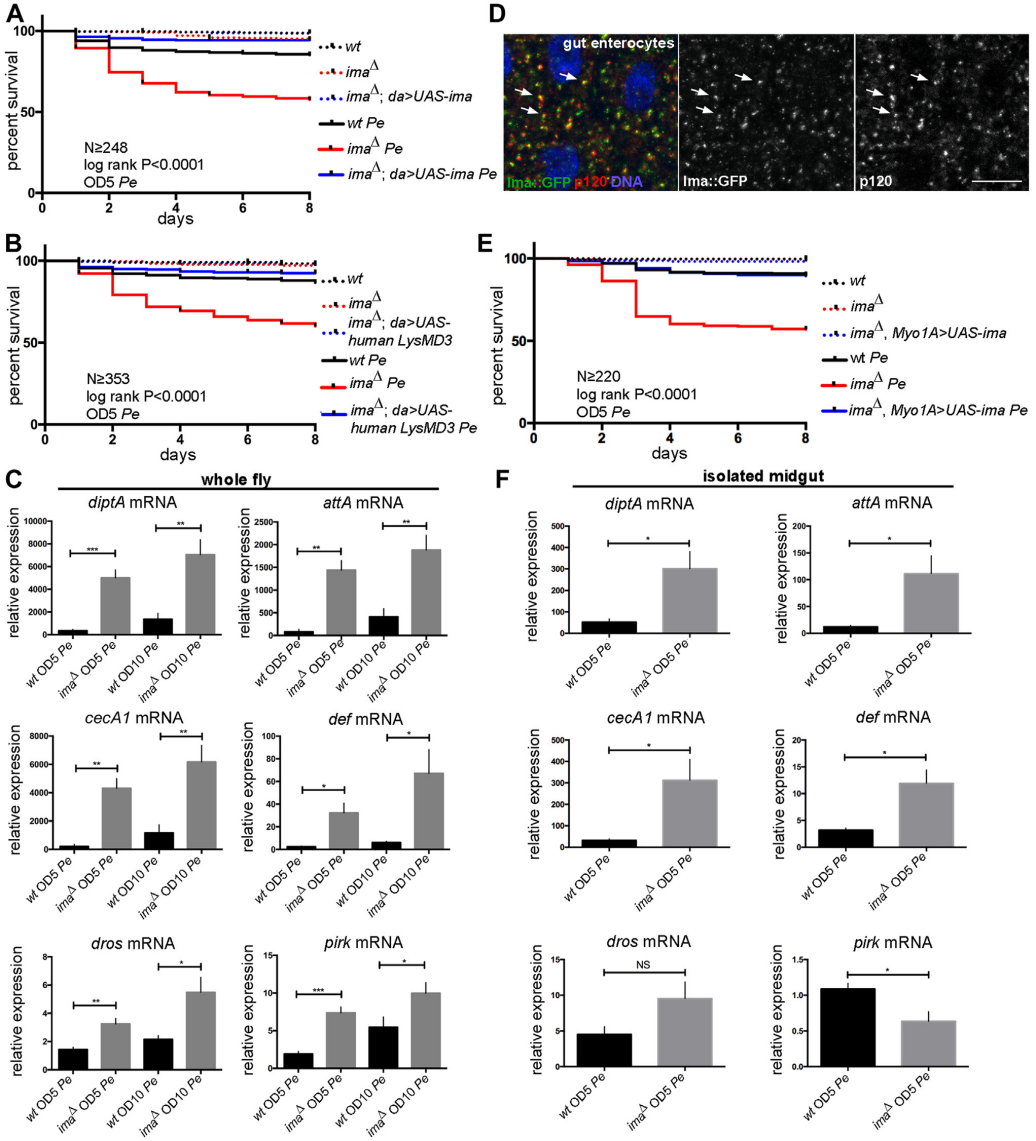
**LysMD proteins protect against gram-negative immune challenge.** A pathogen challenge model was used to test *ima* function in response to *Pseudomonas entomophila* exposure *in vivo*. *A* and *B*, mock treatment, *dotted lines*. Pe treatment, *solid lines*. *w*^*1118*^*, (wt); w*^*1118*^*; ima*^*Δ*^*/ima*^*Δ*^*; da-gal4/+, (ima*^*Δ*^*); w*^*1118*^*; ima*^*Δ*^*/ima*^*Δ*^*; da-gal4/UAS-ima, (ima*^*Δ*^*; da>UAS-ima); w*^*1118*^*; ima*^*Δ*^*/ima*^*Δ*^*; da-gal4/UAS-hLysMD3 (ima*^*Δ*^*; da>UAS-hLysMD3). A*, survival of adult flies homozygous for the *ima*^*Δ*^ mutation in the presence (*blue*) and absence (*red*) of a WT *UAS-ima* transgene under the control of the ubiquitous *da* promoter. n ≥ 248, three independent trials. *p* < 0.0001, log-rank test. *B*, survival of adult flies homozygous for the *ima*^*Δ*^ mutation in the presence (*blue*) and absence (*red*) of a *UAS-hLysMD3* transgene under the control of the ubiquitous *da* promoter. n ≥ 353, three independent trials. *p* < 0.0001, log-rank test. *C*, qRT-PCR was used to measure transcriptional changes in WT and homozygous *ima*^*Δ*^ whole adult flies to a panel of innate immune effector genes 24 h after *Pe* exposure. n ≥ 6. ∗*p* < 0.05, ∗∗*p* < 0.01, ∗∗∗*p* < 0.001, unpaired Student’s *t* test using Welch’s correction. Relative expression indicates normalization to WT mock-treated condition. *D*, confocal microscopy was used to visualize the distribution of Ima-GFP signal in adult midgut enterocytes (*green*, *grayscale middle* panel). Samples were colabeled for the medial Golgi marker P120 (*red*, *grayscale right* panel) and for DNA (*blue*). *E*, survival of adult flies homozygous for the *ima*^*Δ*^ mutation in the presence (*blue*) and absence (*red*) of a *UAS-ima* transgene under the control of the gut enterocyte *Myo1A* promoter. Mock treatment, *dotted lines*. *Pe* treatment, *solid lines*. *w*^*1118*^*, (wt); w*^*1118*^*; ima*^*Δ*^*, Myo1A-gal4/ima*^*Δ*^*(ima*^*Δ*^*); w*^*1118*^*; ima*^*Δ*^*, Myo1A-gal4/ima*^*Δ*^*; UAS-ima/+, (ima*^*Δ*^*, Myo1A>UAS-ima).* n ≥ 220, three independent trials. *p* < 0.0001, log-rank test. *F*, qRT-PCR was used to measure transcriptional changes in isolated adult midguts dissected from WT and homozygous *ima*^*Δ*^ flies on a panel of innate immune effector genes 24 h after *Pe* exposure. n ≥ 6. ∗*p* < 0.05, ∗∗*p* < 0.01, ∗∗∗*p* < 0.001, unpaired Student’s *t* test using Welch’s correction. Relative expression indicates normalization to WT mock-treated condition. Error bars, SE. The scale bar represents 10 μm. ima, immune active; LysMD, Lysin motif domain; qRT-PCR, quantitative reverse transcription PCR.

We first characterized *ima*^*Δ*^ mutants using a Drosophila challenge model of pathogen exposure. *Pseudomonas* entomophila (Pe) is a naturally occurring gram-negative enteric pathogen isolated from wild Drosophila (26). Exposure of WT flies to *Pe* through natural infection leads to a dose-dependent effect on survival (27). Therefore, to determine if *ima*^*Δ*^ flies exhibited a phenotype in this challenge model, adult flies were exposed to Pe infection and survival was scored. Under mock conditions, no difference in survival was observed between WT and homozygous *ima*^*Δ*^ mutant flies (Fig. 3*A*, dotted lines). However, these experiments revealed a marked decrease in the survival of *ima*^*Δ*^ mutants upon *Pe* challenge that was robust in different genetic backgrounds and clearly observable even using low titer *Pe* challenge (Figs. 3*A*, solid lines, S6, *A*–*C*). To further confirm this phenotype, the *ima*^*Δ*^ mutant chromosome was isogenized and then retested in the *Pe* challenge assay under both low and high titer *Pe* exposure (Supporting information, experimental procedures; Fig. S6*D*). Moreover, susceptibility of *ima*^*Δ*^ mutants to *Pe* challenge was rescued by the presence of a WT *ima* transgene (Fig. 3*A*). Notably, the presence of a heterologously supplied WT *ima* transgene was also sufficient to provide protection against immune challenge in this assay, which consistently exceeded that of WT flies (Fig. 3*A*). Similarly, the human *ima* ortholog, *hLysMD3*, was sufficient to confer phenotypic rescue of the *ima*^*Δ*^ mutant in the *Pe* challenge assay (Fig. 3*B*). These data indicate that LysMD-containing proteins encode conserved functions essential for host defense against a gram-negative enteric pathogen.

Natural infection by Pe impacts host viability through effects on the gut epithelial barrier (26). Given that *ima* is necessary to protect against *Pe* challenge and that Ima protein is also present in gut enterocytes (Fig. 3*D*), we next tested if expression of a heterologously supplied WT *ima* transgene under the control of a gut-specific promoter was sufficient to rescue survival. Consistent with its endogenous distribution, expression of *ima* in gut enterocytes is sufficient to completely rescue lethality of the *ima*^*Δ*^ mutant following *Pe* challenge (Fig. 3*E*), although not to the same extent as ubiquitous expression suggesting function in other tissues, as well. Thus, *ima* functions in gut epithelial cells to protect against natural enteric infection by the gram-negative Pe pathogen.

Antimicrobial peptides (AMPs) are key terminal effectors of the humoral innate immune response that are transcribed in response to bacterial challenge (28). Therefore, to extend this characterization of the *ima* loss-of-function phenotype, we used qPCR to directly measure transcription levels using a panel of innate immune system target genes. In each case, loss of *ima* resulted in dysregulation of AMP expression in whole adult flies 24 h following Pe exposure (Fig. 3*C*). Dysregulation of AMP expression was also observed in isolated gut tissue samples derived from *ima*^*Δ*^ mutants (Fig. 3*F*). Taken together, these studies demonstrate that *ima* is necessary to negatively regulate systemic and local AMP expression in response to enteric infection by the gram-negative Pe pathogen.

**Figure 4:**
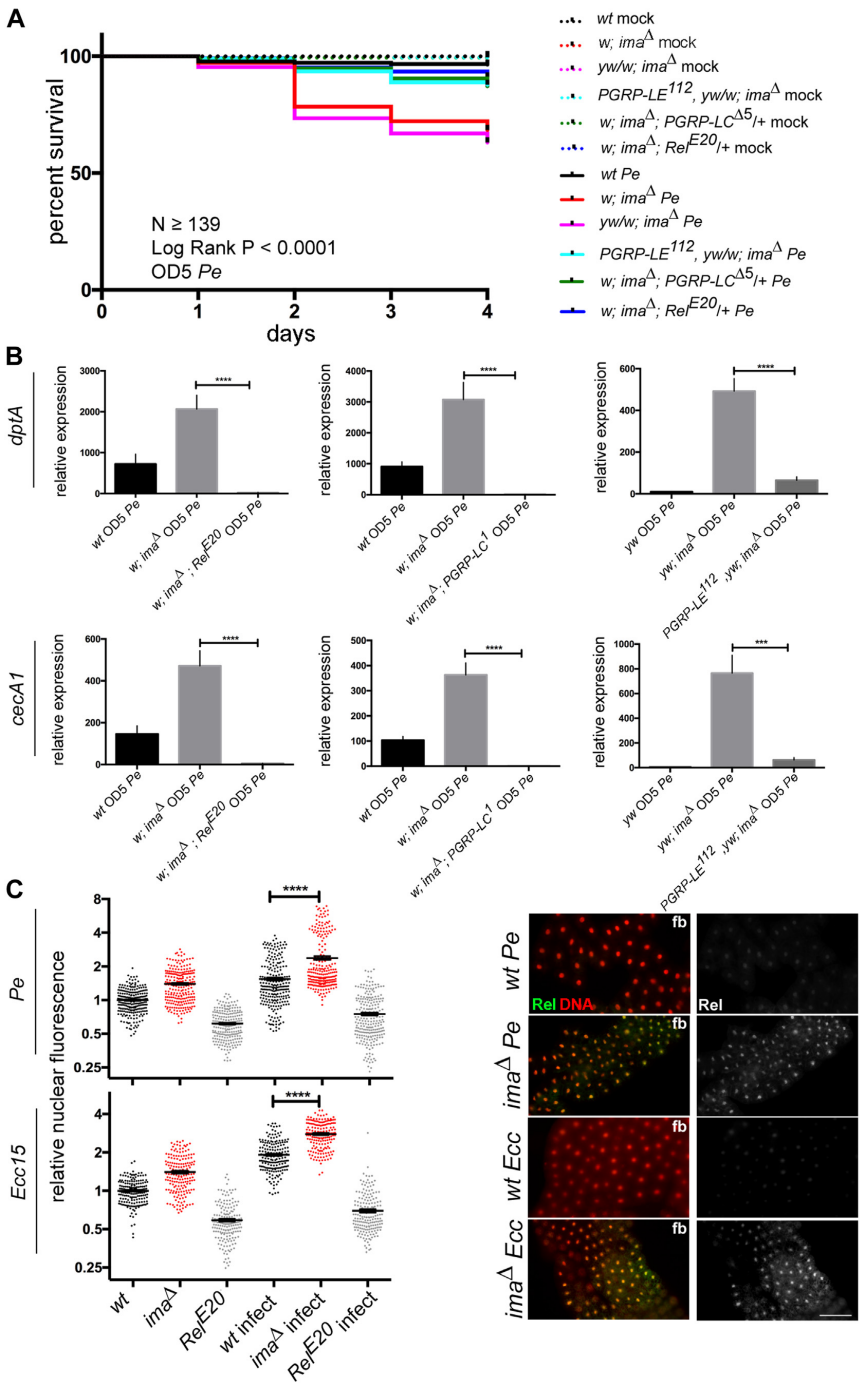
***ima* mutants interact genetically with Imd immune signaling pathway components**. *A* and *B*, pathogen challenge model was used to test genetic interactions between *ima* and Imd pathway components in response to Pe exposure. *A*, survival analysis. *w*^*1118*^*, (wt); w*^*1118*^*; ima*^*Δ*^*/ima*^*Δ*^*(w; ima*^*Δ*^*); y*^*1*^*, w*^*1118*^*/w*^*1118*^*; ima*^*Δ*^*/ima*^*Δ*^*(yw/w; ima*^*Δ*^*); y*^*1*^*, w*^*67C23*^*, PGRP-LE*^*112*^*/w*^*1118*^*; ima*^*Δ*^*/ima*^*Δ*^*(PGRP-LE*^*112*^*, yw/w; ima*^*Δ*^*); w*^*1118*^*; ima*^*Δ*^*/ima*^*Δ*^*; PGRP-LC*^*Δ5*^*/+ (w; ima*^*Δ*^*; PGRP-LC*^*Δ5*^*/+); w*^*1118*^*; ima*^*Δ*^*/ima*^*Δ*^*; Rel*^*E20*^*/*+ (*w; ima*^*Δ*^*; Rel*^*E20*^*/+*). Mock treatment, *dotted lines*. *Pe* treatment, *solid lines*. n ≥ 139, three independent trials. *p* < 0.0001, log-rank test. *B*, AMP analysis. qRT-PCR was used to measure transcriptional changes of the AMPs *dptA* and *cecA1* in adults 24 h after *Pe* exposure. *w*^*1118*^*, (wt); w*^*1118*^*; ima*^*Δ*^*/ima*^*Δ*^*(w; ima*^*Δ*^*); y*^*1*^*, w*^*1118*^*; ima*^*Δ*^*/ima*^*Δ*^*(yw; ima*^*Δ*^*); y*^*1*^*, w*^*67C23*^*, PGRP-LE*^*112*^*; ima*^*Δ*^*/ima*^*Δ*^*(yw, PGRP-LE*^*112*^*; ima*^*Δ*^*); w*^*1118*^*; ima*^*Δ*^*/ima*^*Δ*^*; PGRP-LC*^*Δ5*^*/PGRP-LC*^*Δ5*^*(w; ima*^*Δ*^*; PGRP-LC*^*Δ5*^*); w*^*1118*^*; ima*^*Δ*^*/ima*^*Δ*^*; PGRP-LC*^*1*^*/PGRP-LC*^*1*^(*w; ima*^*Δ*^*; PGRP-LC*^*1*^); *w*^*1118*^*; ima*^*Δ*^*/ima*^*Δ*^*; Rel*^*E20*^*/Rel*^*E20*^ (*w; ima*^*Δ*^*; Rel*^*E20*^)*.* n ≥ 3. ∗∗∗*p* < 0.001, ∗∗∗∗*p* < 0.0001, NS not significant, Student’s *t* test using Welch’s correction. Relative expression indicates normalization to WT mock treated condition. *C*, Relish immunostaining was used to measure Relish nuclear fluorescence relative to untreated *wt* levels in fat bodies (fb) from *wt*, *ima*^*Δ*^ or *Rel*^*E20*^ mutants (*w*^*1118*^; *Rel*^*E20*^) 1 h after systemic *Pe* or *Ecc15* challenge. n ≥ 3. ∗*p* < 0.05, ∗∗*p* < 0.01, ∗∗∗*p* < 0.001, ∗∗∗∗*p* < 0.0001, NS not significant, Student’s *t* test using Welch’s correction. Relative nuclear fluorescence is normalized to untreated WT condition. The scale bar represents 100 μm. AMA, antimicrobial peptides; AMPs, antimicrobial peptides; ima, immune active; Imd, immune deficiency; PGRP, peptidoglycan recognition proteins; qRT-PCR, quantitative reverse transcription PCR.

The host immune response to gram-negative pathogens is mediated by the immune deficiency (Imd) signaling pathway, which can result in death when misregulated (28). In this pathway, peptidoglycan is sensed by the peptidoglycan recognition proteins PGRP-LC and PGRP-LE, and transcriptional responses are mediated by the Relish/NF-kB transcription factor. Therefore, if the response of *ima* mutants to Pe results from Imd pathway hyperactivation as suggested by immune target gene expression, then *ima* mutant phenotypes should be genetically suppressed by simultaneously altering the Imd signaling pathway. To test this prediction directly, the *ima*^*Δ*^ survival phenotype was first analyzed in animals also lacking one copy of established Imd signaling components. This manipulation significantly rescued the survival of *ima*^*Δ*^ mutants in response to *Pe* challenge (Fig. 4*A*). Similarly, the activation of target gene expression seen in *ima*^*Δ*^ mutants was also suppressed following reduction of Imd pathway members (Fig. 4*B*). Finally, nuclear location of the Relish/NF-kB transcription factor, a marker of Imd pathway activation, was found to be elevated in *ima*^*Δ*^ mutants (Fig. 4*C*). Taken together, these findings support the conclusion that Ima normally protects the host against enteric gram-negative challenge by inhibiting Imd pathway activation and thus moderating the tissue damaging effects of AMP misregulation.

## Discussion

The ancient LysMD-containing receptor family is conserved throughout evolution including in humans. LysMD-mediated recognition has been linked to host/pathogen signaling interactions in prokaryotic viruses, prokaryotes, and plants, suggesting that LysMDs are prototypical pattern recognition receptors. Yet, a role for LysMD-containing proteins in animal peptidoglycan sensing has not been directly tested. In this study, we hypothesized that LysMD-containing proteins function as peptidoglycan recognition proteins to regulate immunity in animals. We tested three key predictions of this assertion and demonstrate that Ima, Drosophila LysMD3/4, and its human homolog, hLysMD3, bind peptidoglycan, localize to cell membranes, and promote survival and immune signaling in a pathogen challenge model. Hence, we conclude that LysMD-mediated peptidoglycan recognition is conserved and provides immunity in animals.

There is precedent for peptidoglycan pattern recognition receptors to localize to different subcellular compartments, including the cytoplasm and cell surface. Our results indicate that LysMD receptors such as Ima are among the former class of proteins. Ima interacts genetically with the canonical Imd immune signaling pathway to negatively regulate innate immune signaling through Rel/NF-kB and downstream AMP target genes. Taking the biochemical, localization, and genetic data together suggests several models of Ima function consistent with these findings that may not be mutually exclusive: First, Ima could function as a *bona fide* inhibitory immune receptor to directly regulate host immune responses through a novel pathway. While signal transduction in the *Drosophila* immune system is well described, it is worth noting that a large number of immune responsive genes are regulated independently of the canonical immune signaling pathways (29). Nevertheless, beyond the LysM domain, no additional regions of homology were detected in the *ima* coding region. Second, Ima could function as part of an inhibitory coreceptor complex with previously identified proteins, such as the PGRP family receptors. Such signaling complexes could function to either directly regulate immune activation or act indirectly by influencing the localization, multimerization, or availability of essential immune signaling components. Finally, Ima may function as a competitive agonist (or molecular “sponge”) that does not directly utilize downstream immune effectors but rather functions to buffer levels of free peptidoglycan by sequestering it from interactions with other peptidoglycan sensing systems. According to this model, Ima would protect against lethal tissue damage that would otherwise result from inappropriate innate immune activation. Therefore, it will be important that future studies identify interacting proteins directly associated with LysMD family proteins in animal models.

## Experimental procedures

### *In vitro*–binding assay

Peptidoglycan-binding assays were performed with modifications as previously described (20). Binding reactions with recombinant Ima and hLysM3 proteins were established as follows: 50 μg of ultrapure insoluble *E. coli* K12 peptidoglycan (Invivogen) was mixed in a low protein binding microcentrifuge tube with 1 μg of His-tagged protein in 300 μl volume of 0.1 M phosphate buffer pH 7.0 and incubated overnight at 4 °C.

### Biochemical fractionation

Membrane fractionation assays were performed with modification as previously described (21, 22). Ten female flies were frozen and then homogenized 1 min using a rotary Teflon pestle.

### Microscopy

Tissue samples were processed for immunoelectron microscopy by fixing in 4% formaldehyde in PBS. Negative controls excluded primary antibodies. Whole mount samples for immunostaining was performed using standard methodology. Antisera: mouse anti-p120 Golgi (1:100 Calbiochem); rabbit anti-Relish 130-10080 (1:300, RayBiotech); chicken anti-GFP (1:10,000, Abcam); mouse anti-golgin-84 (1:100, Developmental Studies Hybridoma Bank (DSHB) clone 12-1); guinea pig anti-GMAP (1:2000, DSHB); mouse anti-Hrs (1:100, DSHB clone 27-4); Alexa Fluor–conjugated secondary antibodies (1:2000, Molecular Probes). Whole mount samples were analyzed on a Leica DM5000 compound or Leica TCS SP5 confocal microscope. Images were processed for brightness and contrast in Photoshop CS (Adobe). Fluorescence signal quantitation was performed using ImageJ, and colocalization analysis performed using the JACoP plugin for ImageJ.

### Fly strains

*w*^*1118*^ (BL#3605, control); *isogenized w*^*1118*^ (BL#5905, *w*^*1118iso*^, control); *w*^*1118*^*; ima*^*Δ*^*/CyO, actin-GFP* (this study); *y*^*1*^*, w*^*1118*^*; ima*^*Δ*^*/CyO, actin-GFP* (this study); *w*^*1118*^*; ima*^*Δiso*^*/CyO, actin-GFP* (this study); *w*^*1118*^*; ima::GFP/CyO* (this study); *w*^*1118*^*; UAS-ima* (this study); *w*^*1118*^*; UAS-hLysMD3* (this study); *w*^*1118*^*; da-gal4*; *Myo1A-gal4; w, Relish*^*E20*^ *(loss-of-function mutation in translational start codon); w; PGRP-LC*^*Δ5*^*; w; PGRP-LC*^*1*^*; w; PGRP-LE*^*112*^. Additional information, http://flybase.org.

### Microbial exposure

Natural infections were performed as previously described by exposing adult flies to either *P. entomophila* or *Erwinia carotovora carotovora* 15 (E.cc15) (27). Flies were transferred to standard fly food vials covered with Whatman filter paper and supplemented with 0.2 ml of *Pe* suspension in 5% sucrose or 5% sucrose alone. Following a 24 h exposure, flies were transferred to standard media and their survival was subsequently monitored.

### Quantitative reverse transcription PCR

Tissue was collected from whole adult bodies (n = 10 females per treatment per trial) or from 15 adult guts. *RpL32* was used as a standard unaffected by Pe treatment (27). Transcript levels relative to *rpl32* were calculated using the 2^−Δ*C*T^ method and were normalized to mock-treated WT levels.

## Data availability

All data described in this study are contained within the main manuscript and supporting information.

## Supporting information

This article contains supporting information.

## Conflict of interest

The authors declare that they have no conflicts of interest with the contents of this article.
