# Supplementary material for: Peptidoglycan recognition in Drosophila is mediated by LysMD3/4
Source: J Biol Chem. 2023 Apr 26;299(6):104758. doi: 10.1016/j.jbc.2023.104758 (PMC10220488; doi:10.1016/j.jbc.2023.104758)
Supplement: Supporting information and Figures S1–S6 [file mmc1.pdf]

Peptidoglycan recognition in Drosophila is mediated by LysMD3/4

Mark Snee[1], Jason Wever[1], Jennifer Guyton[1], Ryan Beehler-Evans[1], Christine C. Yokoyama[2], & Craig A. Micchelli[1]*

[1]Department of Developmental Biology,

[2]Department of Pathology and Immunology,

Washington University School of Medicine,

St. Louis, MO 63110, USA

[*]Corresponding author: micchelli@wustl.edu

Supporting Information (SI)

Supplemental Figure S1:

Supplemental Figure S2:

Supplemental Figure S3:

Supplemental Figure S4:

Supplemental Figure S5:

Supplemental Figure S6:

Extended experimental procedures

References

Supplemental Figure S1: Molecular phylogeny and sequence similarity of LysMD proteins

A

B

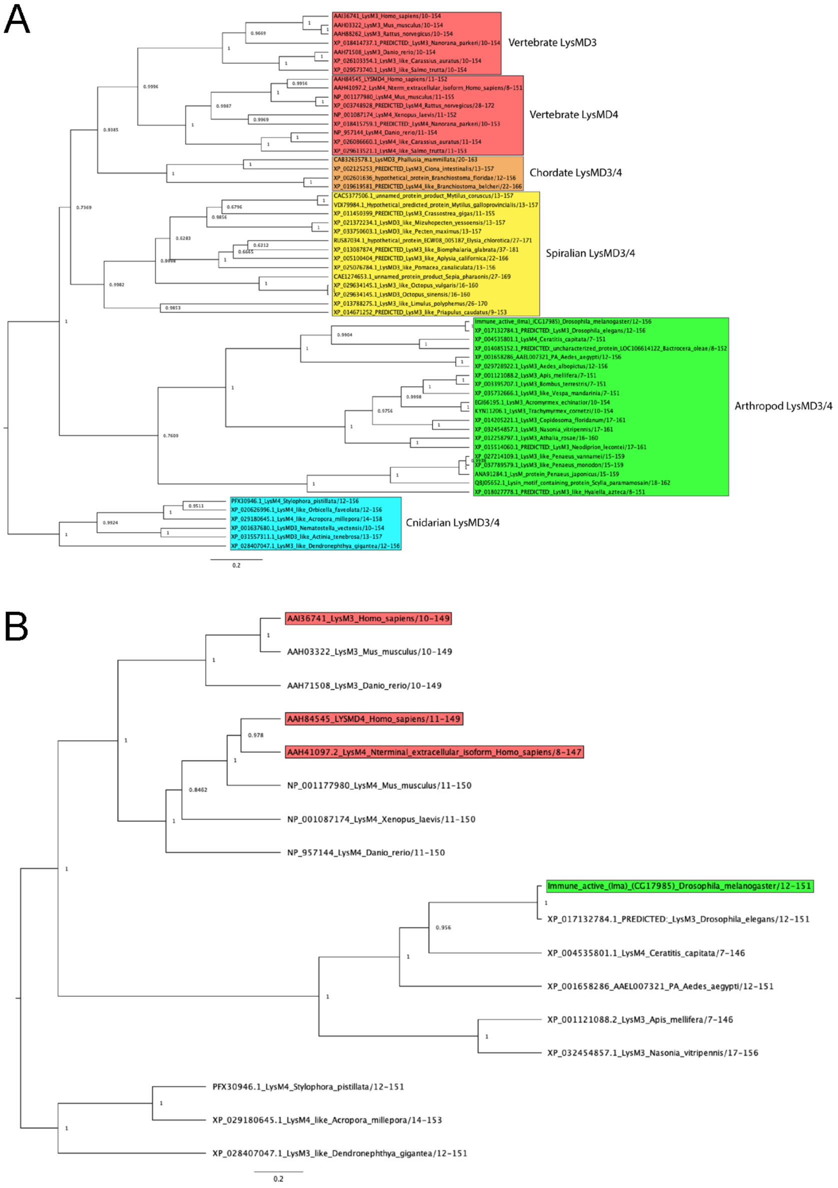

C

Dmel LysM3/4 BLAST Sequence Comparisons

Sequence Compared To	Max Score	Total Score	Coverage (%)	E Value	% ID
HsLysMD3 full length [AAI36741]	50.1	66.2	74	1.00E-11	23.65
HsLysMD4 full length [AAH84545]	48.1	65.8	72	3.00E-11	23.79
HsLysMD4 (N-term extracellular) full length [AAH41097.2]	49.7	49.7	75	1.00E-11	21.84
HsLysMD3 LysM domain [AAI36741]	50.8	50.8	82	2.00E-16	51.16
HsLysMD4 LysM domain [AAH84545]	42	42	90	1.00E-12	38.3
HsLysMD4 (N-term extracellular) LysM domain [AAH41097.2]	34.3	34.3	76	1.00E-09	38.3

Supplemental Figure S1: Molecular phylogeny and sequence similarity of LysMD proteins

A,B) Cladograms of LysMD3 and LysMD4 protein phylogenies using Bayesian analysis. Node labels indicate posterior probability. Scale bar = number of substitutions per amino acid residue. A) LysMD3 and LysMD4 from representative species in Vertebrata, Chordata, Spiralia, Arthropoda, and Cnidaria. Consensus tree resolved with the expected topology in relation to known evolutionary relationships, using cnidarian sequences as the tree root. Six major clades were identified, from bottom to top: blue, Cnidarian LysMD3/4; green, Arthropod LysMD3/4; yellow, Spiralian LysMD3/4; orange, Chordate LysMD3/4; red, Vertebrate LysMD3 and Vertebrate LysMD4. In the genomes of all sampled animals other than vertebrates (including chordates), there exists a single LysMD3- or LysMD4-like gene, which also holds true for the most basal sampled clade, Cnidaria. This suggests that distinct LysMD3 and LysMD4 proteins are the result of a duplication event in vertebrates. Thus, we use a naming convention in which all non-vertebrate LysMD3 and LysMD4 proteins are designated “LysMD3/4,” indicative of the characteristics of the last common metazoan ancestor. B) LysMD3/4 proteins from representative species in Vertebrata, Insecta, and Cnidaria. Notably, phylogenetic analysis suggests more similarity between *Drosophila* Ima and human LysMD3 than to either human LysMD4 isoform, reflected by a lower number of substitutions per amino acid. Red, Human LysMD3 and LysMD4; green, *Drosophila melanogaster* Ima. C) The full-length protein sequence of *Drosophila* Ima was compared by BLAST analysis using blastp to all full-length human LysMD3 and LysMD4 proteins. Human LysMD4 has numerous isoforms and comparison was done to two protein isoforms representative of all those from similar transcripts (NCBI accession numbers AAH84545 and AAH41097.2). Full-length *Drosophila* Ima showed slightly more similarity to human LysMD3 than to either human LysMD4 isoform. BLAST analysis comparing the LysM domains alone revealed a much more significant similarity between *Drosophila* Ima and human LysMD3. The above combined phylogenetic and sequence analysis data indicates *Drosophila* Ima and human LysMD3 are orthologs.

Supplemental Figure S2: LysMD proteins differentially bind glycans

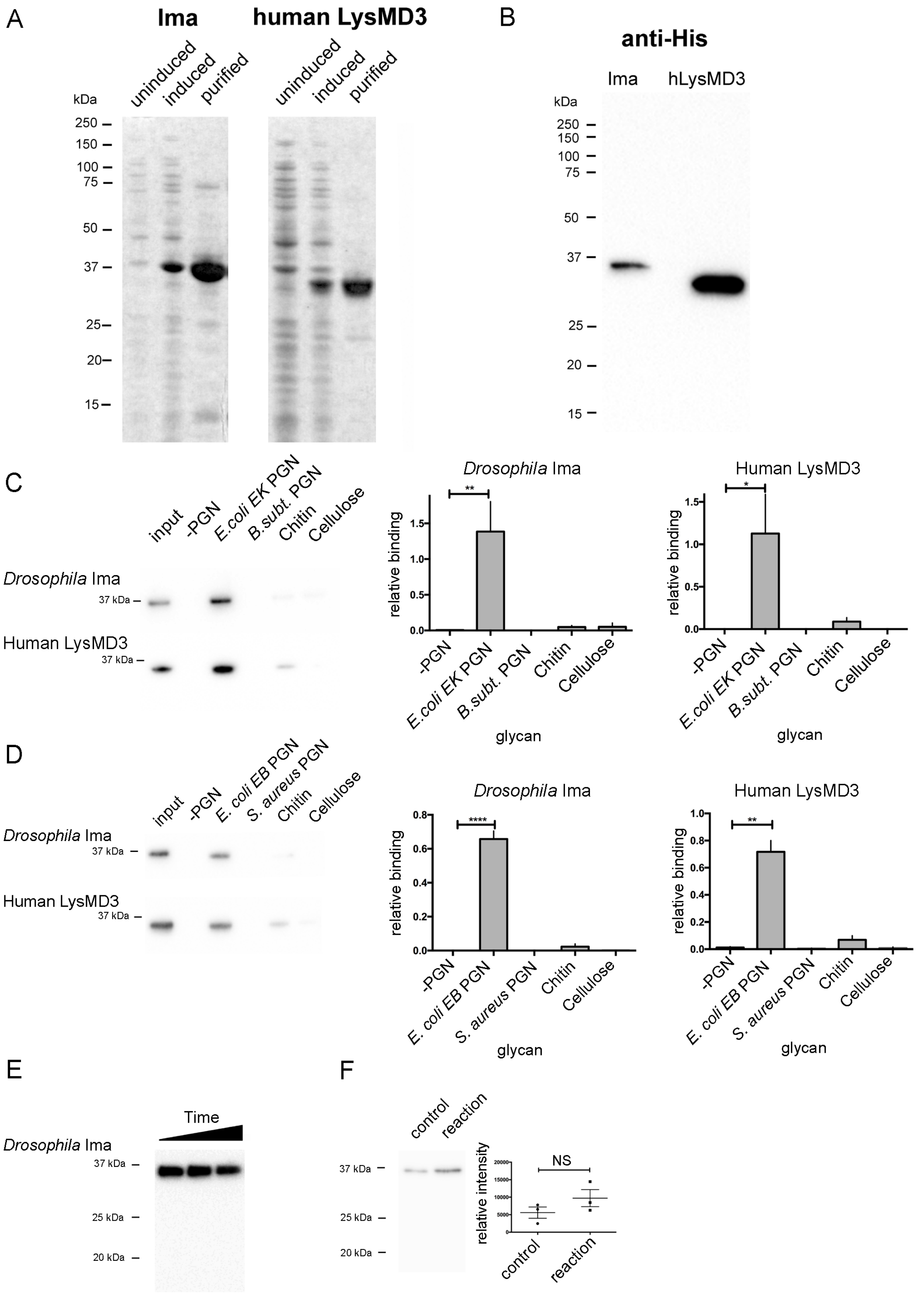

Supplemental Figure S2: LysMD proteins differentially bind glycans

A) Coomassie-stained SDS-PAGE gels showing total bacterial protein from *E. coli* without IPTG (uninduced), with IPTG induction (induced), or affinity purified protein eluted from Ni-NTA resin for the expression and purification of 6XHis-tagged Ima (left panel) or human LysMD3 (right panel). B) Anti-His Western blot showing specific detection of purified 6XHis-tagged Ima and human LysMD3. C,D) An affinity-binding assay was used to measure the interaction between recombinant LysMD-containing proteins and panels of structurally distinct glycans derived from *E. coli* strain *K12* (*E. coli EK*), *E. coli* strain *O111:B4* (*E. coli EB*), *Bacillus subtilis* (*B. subtilis*), *Staphylococcus aureus* (*S. aureus*), chitin, and cellulose. Western blots were probed with anti-His antibodies to detect 6XHis-tagged proteins and signal intensity was quantified. 2% of the total 6XHis-tagged protein present in each binding reaction was directly loaded on the gel alone, as a positive control for detection of 6XHis-tagged protein on the blot (Input). C) 6XHis-Ima or human LysMD3 were incubated in the absence of PGN (-PGN), or in the presence of *E. coli EK* PGN, *B. subtilis.* PGN, chitin, and cellulose; n=3 independent trials. Values normalized to input. D) 6XHis-Ima or human LysMD3 was incubated in the absence of PGN (-PGN), or in the presence of *E. coli EB* PGN, *S. aureus*. PGN, chitin, and cellulose; n=3 independent trials. Values normalized to input. E) 6XHis-Ima was incubated in the presence or absence of PGN (+/-PGN). The effect of increasing wash time prior to elution on the extent of PGN binding was measured; n=3 independent trials. Same gel as the one presented in Fig.1F. No protein degradation products can be detected on the overexposed gel. F) Stability of 6XHis-Ima measured in reaction buffer. The reaction was assembled; split into two samples, one that was shifted to -20°C overnight (control) and the other incubated at 4°C overnight (reaction); and then compared by Western blotting; n=3 independent trials. Relative intensity is in arbitrary fluorescence units. * $P < 0.05$, ** $P < 0.01$, **** $P < 0.0001$, NS, not significant; unpaired Students t-test; Error bars, SE.

Supplemental Figure S3: LysMD proteins localize to cell membranes

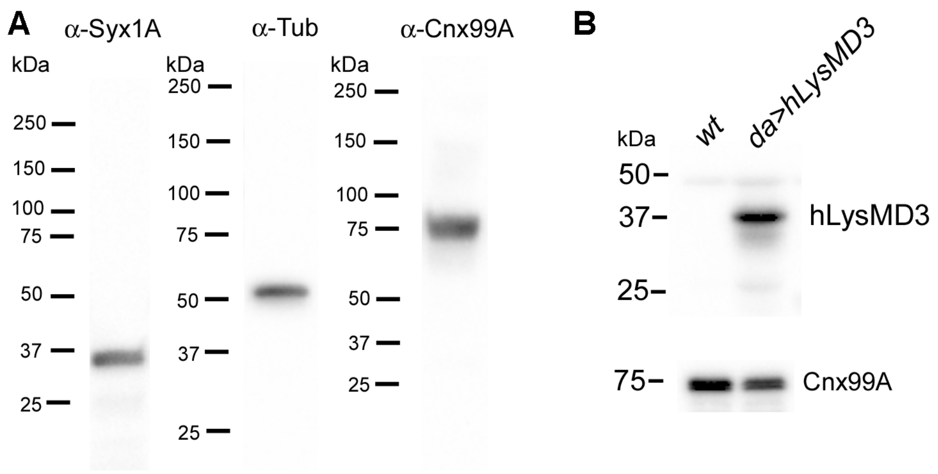

Supplemental Figure S3: LysMD proteins localize to cell membranes

A) Western blots of wild type adult whole fly lysates for Anti-Syx1A, anti-Tubulin, and anti-Cnx99A show a single stained band of the expected mass. B) Western blot analysis using whole adult lysates from wild type and *da-gal4>UAS human LysMD3* flies were probed with anti-hLysM3 antibodies. Cnx99A was used as a positive control.

Supplemental Figure S4: Correlation between Ima-GFP and endomembrane markers

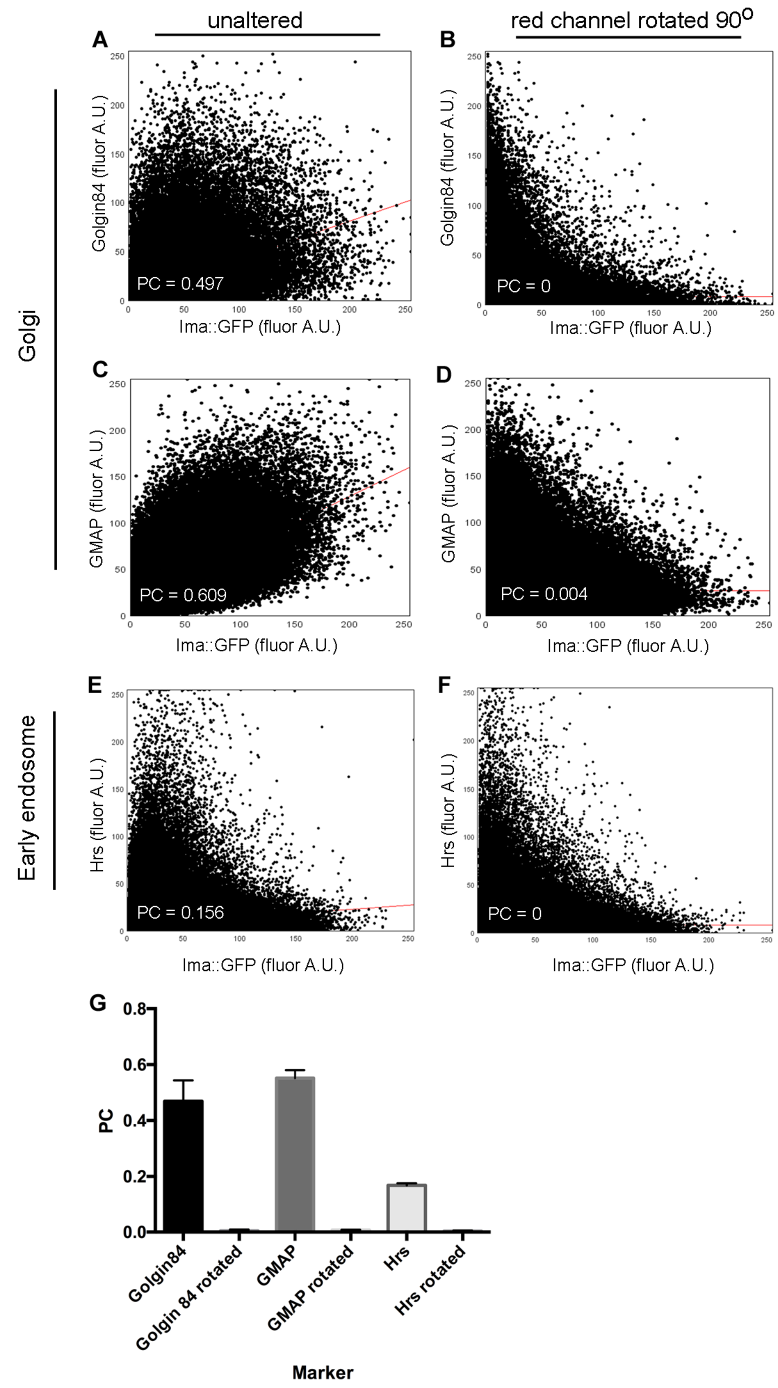

Supplemental Figure S4: Correlation between Ima-GFP and endomembrane markers

A-F) Scatter plots show the extent of Ima-GFP colocalization with endomembrane markers following double immunolabeling in data obtained from individual confocal micrographs. Pearson's coefficient (PC). A) Fluorescence of Ima-GFP plotted against the Golgi rims associated marker Golgin84. The PC value indicates a moderate correlation between these markers. B) Negative control. Fluorescence of Ima-GFP plotted against the Golgi rims associated marker Golgin84, when the Golgin84 channel was arbitrarily rotated 90°. C) Fluorescence of Ima-GFP plotted against the cis-Golgi associated marker GMAP. The PC value indicates a moderate correlation between these markers. D) Negative control. Fluorescence of Ima-GFP plotted against the cis-Golgi associated marker GMAP, when the GMAP channel was arbitrarily rotated 90°. E) Fluorescence of Ima-GFP plotted against the early endosome associated marker Hrs. The PC value indicates no correlation between these markers. F) Negative control. Fluorescence of Ima-GFP plotted against the early endosome associated marker Hrs, when the Hrs channel was arbitrarily rotated 90°. G) Quantitation. Comparison of PC values associated with different endomembrane markers, n=3 for each condition. Error bars, SE.

Supplemental Figure S5: Generation of an *ima* mutant

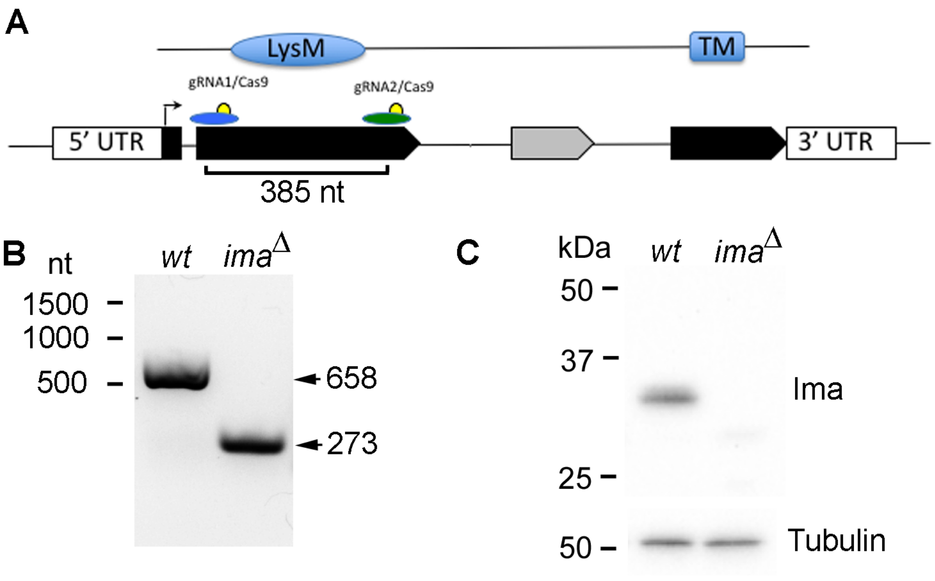

Supplemental Figure S5: Generation of an *ima* mutant

A) Genomic organization of the *lysMD3/4* locus (CG17985) and CRISPR/Cas9 strategy targeting the LysM domain. A pair of guide RNAs was designed to produce a 385 nucleotide deletion at the locus. B) A PCR screening assay was used to identify individuals carrying the engineered deletion. The presence of a short 273 nt band was diagnostic of a deletion at the locus. C) Western blot analysis using whole adult lysates from wild type and ima^{Δ} mutant flies were probed with anti-Ima and anti-Tubulin antibodies, as a positive control.

Supplemental Figure S6: Effect of genetic background, pathogen titer and isogenization on the *ima* survival phenotype following *Pe* challenge

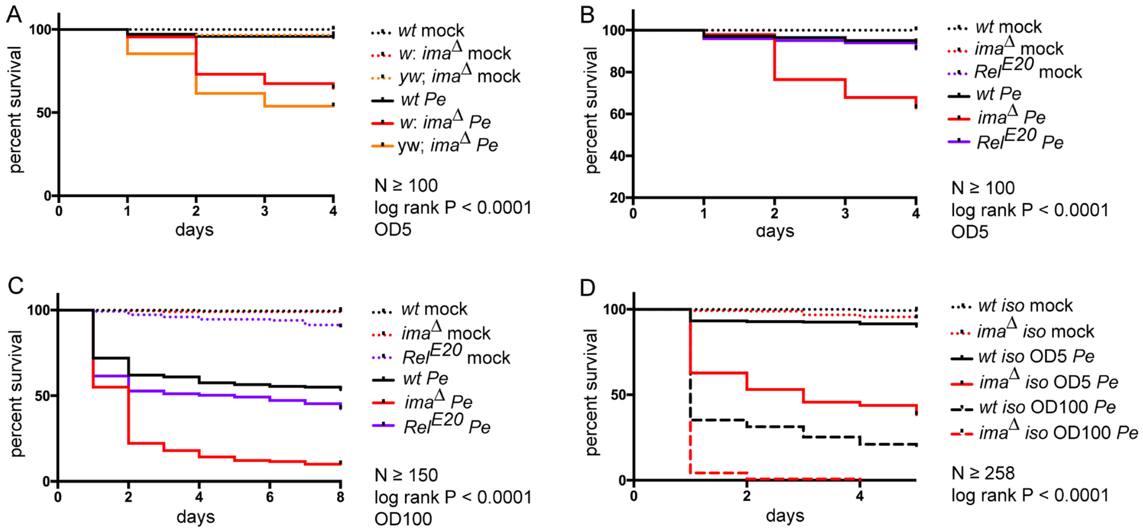

Supplemental Figure S6: Effect of genetic background, pathogen titer and isogenization on the *ima* survival phenotype following *Pe* challenge

A-D) Adult flies were challenged with *Pe* and survival was measured. Mock treatment, dotted lines. *Pe* treatment, solid lines. OD as indicated (OD5, low-titer challenge; OD100, high-titer challenge). *w^{1118}, (wt); w^{1118}; ima^{Δ}/ ima^{Δ}, (w; ima^{Δ}); y^{1}, w^{1118}; ima^{Δ}/ ima^{Δ}, (yw; ima^{Δ}); w^{1118}; Rel^{E20}/ Rel^{E20}, (Rel^{E20}), w^{1118}, (wt iso); w^{1118}; $ima^{\Delta iso}$/ $ima^{\Delta iso}$, ($ima^{\Delta iso}$).* A) Effect of genetic background on the *ima* mutant phenotype (i.e. *w*, *yw*). Survival of the *ima^{Δ}* mutant does not differ significantly from wild type under mock treatment conditions. *Pe* treatment results in a significant difference in survival of both *w*; *ima^{Δ}* and *yw*; *ima^{Δ}* mutants compared to wild type at OD5. n ≥ 100, three independent trials. $P < 0.0001$, log rank test. B) Effect of low-titer (OD5) *Pe* challenge on the *ima* mutant phenotype. Survival of wild type flies does not differ significantly from *ima^{Δ}* mutants under mock treatment conditions. Survival of wild type flies differs significantly from *ima^{Δ}* mutants at OD5. n ≥ 100, three independent trials. $P < 0.0001$ log rank test. Note, *Rel^{E20}* mutants differ significantly from *ima^{Δ}* mutants at OD5. C) Effect of high-titer (OD100) *Pe* challenge on the *ima* mutant phenotype. Survival of wild type flies does not differ significantly from *ima^{Δ}* mutants under mock treatment conditions. Survival of wild type flies differs significantly from *ima^{Δ}* mutants at OD100. n ≥ 150, three independent trials. $P< 0.0001$ log rank test. Note, *Rel^{E20}* mutants differ significantly from *ima^{Δ}* mutants at OD100. D) Effect of isogenizing the *ima* mutant. Isogenized *ima^{Δ}* mutant flies exhibit a survival phenotype that becomes more severe with increasing *Pe* exposure (compare solid and dashed red lines). n ≥ 258, three independent trials. $P < 0.0001$, log rank test. Note, isogenizing the *ima^{Δ}* mutant resulted in a slightly stronger phenotype, suggesting the presence of one or more suppressors in the original parental strain (compare to red lines in B,C).

Extended experimental procedures

Fly strains
w^{1118} (BL#3605, control); *isogenized w^{1118}* (BL#5905, *$w^{1118iso}$*, control); *w^{1118}; ima^{Δ}/CyO, actin-GFP* (this study); *y^1, w^{1118}; ima^{Δ}/CyO, actin-GFP* (this study); *w^{1118}; $ima^{\Delta iso}$/CyO, actin-GFP* (this study); *w^{1118}; ima::GFP/CyO* (this study); *w^{1118}; UAS-ima* (this study); *w^{1118}; UAS-hLysMD3* (this study); *w^{1118}; da-gal4; Myo1A-gal4; w, $Relish^{E20}$; w; $PGRP\text{-}LC^{\Delta5}$; w; $PGRP\text{-}LC^1$; w; $PGRP\text{-}LE^{112}$. Additional information, http://flybase.org.*

CRISPR/Cas9
ima loss-of-function mutants were generated by creating the guide RNA plasmid *ima* pCFD4, which produces guide RNAs (targeting genomic regions GATCTGCTCCCTCTGGCCAG and GGTAAATGCTTCAGGCGCCG) designed to delete the conserved lysin motif domain of *ima*. *ima* pCFD4 was designed and synthesized following the standard "Cloning two gRNAs into plasmid *pCFD4*" protocols outlined on the Bullock lab database (www.crisprflydesign.org/gRNA-expression-vectors/) and as previously described[1,2]. PCR amplification of the *ima*-specific fragments for cloning was performed using Q5 polymerase, 10 ng/µl of pCFD4 as a template, 3.3% DMSO and 100 nM final concentration of ima pCFD4 fwd and ima pCFD4 rev primers. PCR was performed 98°C for 2 min, 16 cycles of 98°C for 10 s, 55°C for 20s (ramping to 63 °C at 0.5 °C/cycle), and 72 °C for 15 s, then 15 cycles of 98°C for 10 s and 72°C for 25 s, with final extension of 72°C for 5 min. Plasmid assembly was performed using Gibson Assembly (NEB) following the manufacturer's protocol.

The *ima* pCFD4 plasmid was injected into *nos-Cas9* transgenic embryos to induce Cas9-mediated germline deletion of the LysM domain of *ima*. Embryo injection and subsequent screening for survival to adulthood was performed by BestGene Inc (Chino Hills, CA). All viable F0 adults were backcrossed to double balancer chromosomes to remove *nos-Cas9* from germline and to isolate and stabilize potential *ima^{Δ}* mutations. All potential mutants were screened by PCR, using the *ima* screen fwd and *ima* screen rev primers. Several independent *ima^{Δ}* deletion mutants were obtained. Three independent mutants that caused a deletion of the lysin motif domain and a frame shift mutation were analyzed further and had similar effects on the transcriptional response to *Pe* infection. One of these lines, *$ima^{\Delta12A1}$*, (ATCTGCTCCC[deletion]CACGAGAAAC), referred to here as *ima^{Δ}* was selected for further analysis. Additionally, *$ima^{\Delta12A1}$* was backcrossed to isogenic *w^{1118}* flies (BL#5905, *$w^{1118iso}$*) for 8 generations, while tracking the mutant via PCR to generate the isogenized *$ima^{\Delta iso}$* strain. The *$ima^{\Delta iso}$* strain exhibited a more severe phenotype following pathogenic challenge suggesting the presence of one or more suppressors in the original parental genetic background.

An *in vivo* GFP tagged form of the *ima* gene was generated using CRISPR/Cas9 to delete the regions between the *ima* stop codon and an intron of the gene

immediately 3’ of *ima* gene, *CG43340*. Homology directed repair was used to replace these sequences with those containing C terminal GFP tag in the *ima* gene. An *ima::GFP* guide RNA plasmid in pCFD4 (targeting genomic regions AGGTTCTCTAAAGAGCTAAC and AGTAGTTGGGTAATGTCTGC) was created using the primers MsGFPguide1 and MsTMguide2. A homology directed repair template plasmid was generated by using Gibson assembly to clone a left homology arm (primers LysGFPvivoLH1 and LysGFPvivoLH2), GFP coding sequence (primers LysGFPvivoLH3 and LysGFPvivoLH4), and 3’ *ima* gDNA sequences (primers LysGFPvivoLH5 and LysGFPvivoLH6) into the SacII site of the pHD-DsRed-attP plasmid. The right homology arm was cloned by using Gibson assembly to insert a right homology arm PCR product (primers GFPvivoRH1 and GFPvivoRH2) into the AvrII site of pHD-DsRed-attP. The *ima*::GFP guide RNA plasmid and *ima*::GFP homology repair plasmid were co-injected into *nos-Cas9* embryos (Bestgene Inc.), progeny were crossed to balancer stocks, and 3XP3 DsRed positive lines were isolated and confirmed to have the desired insertion. The 3XP3 DsRed marker sequences flanked by loxP sites were removed by crossing to P{Crey}1b flies and selecting progeny that retained GFP fluorescence but lacked 3XP3 DsRed.

Transgenes

The *UAS-ima* transgene UFO6039 is a C terminally Flag/HA tagged *ima* construct that was obtained from the BDGP via the DGRC. The *UAS human LysMD3* expression construct was generated by PCR using primer HuLys3F and HuLys3R using the human *LysMD3* encoding plasmid HsCD000082017 (DNASU plasmid repository) as a template, and using Gibson assembly (NEBuilder, NEB) to insert the PCR product into KpnI and XbaI digested pJFRC28. The *UAS-ima* and *UAS-hLysMD3* constructs were integrated into the attP2 site via phiC31 integrase by injecting constructs the fly stock y^1, w^{67c23}; *P{y[+t7.7]=CaryP}attP2* (Bestgene Inc.). Positive transformants were recovered and crossed into a w^{1118} background.

Vectors, cloning & cDNAs

The Ima protein expression construct spans sequences 1-705 nt of the *ima* coding sequence (cDNA LD36653) and contains the N-terminal regions of Ima including the Lysin motif, but excludes regions C terminal to the start of the transmembrane domain. A gBlocks DNA fragment (Integrated DNA Technologies) containing these sequences was inserted into the HindIII site of pET29a using Gibson assembly.

The Ima expression construct was modified via point mutagenesis to produce the Ima N85A protein expression construct, which contains the mutation AAC to GCC (N85A). Mutagenesis was performed using primers containing the N85A mutation (N85ultraguiderev, N85APetrightfwd, N85ultraguidefwd, and N85APetleftrev) to generate overlapping PCR products containing the desired mutation. PCR products were inserted into the HindIII site of pET29a via Gibson assembly.

The human LysMD3 expression construct spans sequences 1-645 nt of the *hLyMD3* coding sequence and contains the N terminal region of LysMD3 including the Lysin motif, but excludes regions C terminal to the start of the transmembrane domain. PCR was performed using the primers hLMD3petfwd and hLMD3petrev2 using a human *LysMD3* plasmid (HsCD000082017, DNASU) as a template. The PCR product was inserted into the HindIII site of pET29a using Gibson assembly.

In vitro affinity binding assay
Peptidoglycan binding assays were performed with modifications as previously described[3]. Initial binding reactions with Ima and hLysM3 proteins were established as follows: 50 µg of ultrapure insoluble *Escherichia coli* K12 peptidoglycan (Invivogen) was mixed in a low protein binding microcentrifuge tube with 1 µg of His-tagged protein in 300 µl volume of 0.1 M phosphate buffer pH 7.0 and incubated overnight at 4°C. Insoluble peptidoglycan was pelleted at 14000 rpm, the supernatant was removed and the pellet was resuspended in 1 ml of phosphate buffer. The contents were washed two additional times in this way, and transferred to a new tube after each wash. Bound proteins in the pellet were eluted with 30 µl of SDS loading buffer and were analyzed via anti-His staining on Western blot. Association time course studies were performed by first establishing the peptidoglycan/protein reaction mix described above using *Escherichia coli* 0111:B4 peptidoglycan (Invivogen) and incubating for 1, 5, or 15 min, while rocking at room temperature. Dissociation studies were performed by first establishing binding reactions with *Escherichia coli* 0111:B4 peptidoglycan and incubating for 10 min while rocking at room temperature. Following the first wash, peptidoglycan/protein complexes were resuspended in 1.5 ml of buffer and allowed to dissociate for 10 min or overnight at 4 °C before pelleting peptidoglycan and eluting bound proteins with SDS loading buffer. Competition assays were performed by adding 0, 25 or 50 µg of soluble ultrapure *Escherichia coli* K12 (Invivogen) to binding assays containing *Escherichia coli* 0111:B4 peptidoglycan (Invivogen) and incubating for 10 min, while rocking at room temperature. Comparison of wild type and Ima mutant protein binding were performed by establishing binding reactions with *Escherichia coli* 0111:B4 peptidoglycan (Invivogen) and incubating for 10 min, while rocking at room temperature. Side-by-side comparisons of Ima binding to the glycan panels were performed as above with 50 µg of *Escherichia coli* K12 (Invivogen), *Escherichia coli* 0111:B4 (Invivogen), *Bacillus Subtilis* (Sigma), or *Staphylococcus Aureus* (Sigma) PGN or with 50 µg of cellulose (MP Biomedicals) or chitin (Alfa Aesar). Each binding experiment performed used a single batch of commercially sourced ligand to minimize variation within experimental cycles.

DNA isolation
gDNA samples for PCR were generated using either using a modification of a single fly gDNA protocol or the quick DNA miniprep plus kit (Zymo Research). For the single fly protocol, two flies were homogenized in 100 µl of 10 mM Tris

pH 8.0, 1 mM EDTA, 25 mM NaCl, 200 µg/ml Proteinase K. The homogenate was incubated at 37°C for 30 min then 95°C for 2 min.

Western blots
Samples were run on SDS PAGE gels, transferred to PVDF, blocked 1 h in 5% skim milk powder in PBS 0.1% Tween then incubated with primary antibodies mouse anti-6X His (1:2000 overnight, ThermoFisher clone HIS.H8), rabbit anti-Ima (1:5000 1 h, this study), mouse anti-beta Tubulin (cytoplasmic marker; 1:100 overnight, DSHB clone E7), mouse anti-Syx1A (membrane marker; 1:100 overnight, DSHB clone 8C3), Rabbit anti-hLysMD3 (1:5000, Proteintech), mouse anti-Cnx99A (membrane marker; 1:100 overnight, DSHB clone 6-2-1). Membranes were washed and incubated with secondary antibodies anti-mouse HRP (1:5000 1 h, Invitrogen) or anti-rabbit HRP (1:5000 1 h, Cell Signaling technology) before imaging with Clarity ECL substrate (Bio-Rad) and ChemiDoc MP imaging system (Bio-Rad).

Protein purification
Rosetta 2 (DE3) cells (Sigma) transformed with *ima* or human *LysMD3* expression constructs were induced with 0.5 mM IPTG for 4 h at 30°C. Cells were pelleted, and resuspended in PBS 0.1% Triton, with 1 mg/ml lysozyme added. The cell suspension was incubated on ice 30 min prior to lysis via sonication. Lysates were cleared via centrifugation and His tagged proteins were batch purified using HisPur Ni-NTA resin (Thermo Scientific). Bound proteins were eluted with 250 mM imidazole and dialyzed against three changes of PBS overnight. Protein concentrations were determined using Bradford Assay reagent (Thermo Scientific) and BSA standards.

Biochemical fractionation
Membrane fractionation assays were adapted with modifications as previously described[4,5]. Ten female flies were frozen then homogenized 1 min using a rotary teflon pestle in 200 µl of 50 mM Tris-HCl, pH 7.5, 25% Sucrose, 5% glycerol, 10 mM EDTA, 1 mM DTT plus protease inhibitors. The homogenate was centrifuged at 2000 g for 3 min, the supernatant was removed, diluted with an equal volume of water and separated into two low protein binding microcentrifuge tubes. Homogenates were centrifuged at 20000 g for 2 hours at 4°C and the supernate was removed, precipitated using a methanol/chloroform method, resuspended in SDS loading buffer, and kept as the cytoplasmic fraction. The pellet was washed with 200 µl of 20 mM Tris-HCl, pH 7.5, 1 mM EDTA, centrifuged at 20000 g for 1 hour, resuspended in SDS loading buffer, and kept as the crude membrane fraction.

Antisera production
Purified His-tagged Ima protein (described above) was used as an antigen to produce anti-Ima antisera in rabbits (Pocono Farms and Laboratory).

Primers

HuLys3F	CGGCCGCGGCTCGAGGGTACaacttaaaaaaaaaaatcaaaATGGCAGGGAGGCATCAGAA
HuLys3R	GATTTGTTATTTTAAAAACGATTCATTTGTTTCCTGTTGAGCAGCAG
hLMD3petfwd	GAATTCGAGCTCCGTCGACATATGGCAGGGAGGCATCAGAA
hLMD3petrev2	GGTGCTCGAGTGCGGCCGCACCTCCCCAGTCTGCTCCATAATAG
ima pCFD4 fwd	TATATAGGAAAGATATCCGGGTGAACTTC[GATCTGCTCCCTCTGGCCAG]GTTTTAGAGCTAGAAATAGCAAG
ima pCFD4 rev	ATTTTAACTTGCTATTTCTAGCTCTAAAAC[CGGCGCCTGAAGCATTTACC]GACGTTAAATTGAAAATAGGTC
ima screen fwd	CCAACAACAACACAATCAGGA
ima screen rev	CAGCACTGACCTTTCTTCACC
N85APetleftrev	GATTTCATTTTCGCGGTCGATCTTGgcCAGGCGCTTGATGTCCGCCA
N85APetrightfwd	TGGCGGACATCAAGCGCCTGgcCAAGATCGACCGCGAAAATGAAATC
MsGFPguide1	TATATAGGAAAGATATCCGGGTGAACTTC[gttagctctttagagaacct]GTTTTAGAGCTAGAAATAGCAAG
MsTMguide2	ATTTTAACTTGCTATTTCTAGCTCTAAAAC[AGTAGTTGGGTAATGTCTGC]GACGTTAAATTGAAAATAGGTC
LysGFPvivoLH1	tcttgcatgctagcggccgcTGAGCTGGTGCAAAAGTACG
LysGFPvivoLH2	AACAGTTCTTCACCTTTGGACATggACTGGGCACTATGGTTGTGA
LysGFPvivoLH3	TCACAACCATAGTGCCCAGTccATGTCCAAAGGTGAAGAACTGTT
LysGFPvivoLH4	GGGTGCAGACATTACCCAACTTTACTTGTAGAGCTCATCCATGCC
LysGFPvivoLH5	GGCATGGATGAGCTCTACAAGTAAAGTTGGGTAATGTCTGCACCC
LysGFPvivoLH6	GCAGGTGtgcatatgtccgcccttggtcgagctattactattctac
GFPvivoRH1	tctccatgcataaggcgcgcttctctaaagagctaacGTTTCGAG
GFPvivoRH2	AGCctcgagctgcagaaggcTCCAAGTCCTGGATGAGTCC
rpl32FWD	GACGCTTCAAGGGACAGTATCTG
rpl32REV	AAACGCGGTTCTGCATGAG
dptAFWD	GCTGCGCAATCGCTTCTACT
dptAREV	TGGTGGAGTGGGCTTCATG
attAFWD	CACAACTGGCGGAACTTTGG
attAREV	AAACATCCTTCACTCCGGGC
cecA1FWD	AAGCTGGGTGGCTGAAGAAA
cecA1REV	TGTTGAGCGATTCCCAGTCC
defFWD	ATCGCTTTTGCTCTGCTTGC
defREV	ATCCTCATGCACCAGGACATG
drosFWD	CGTGAGAACCTTTTCCAATATGATG
drosREV	TCCCAGGACCACCAGCAT
pirkFWD	TCGATCGAAAAACGCCAAGC
pirkREV	TGCTCACGTTGATGGCATTC

PCR

PCR was performed using Q5 DNA or Taq polymerase and the appropriate template and primers.

qRT-PCR

Tissue was collected from whole bodies (n=10 females per treatment per trial) or from 15 adult guts and analyzed from at least 3 separate infection trials. Total RNA was extracted using the DirectZol RNA Miniprep Kit (Zymo) according to the

manufacturer's instructions. The quality and quantity of RNA were assessed using a NanoDrop 1000 Spectrophotometer (Thermo Scientific). cDNA was synthesized from 1 μg RNA using the High-Capacity cDNA Reverse Transcription Kit according to the manufacturer's protocol (Applied Biosystems). cDNA was subsequently treated with RNase H (NEB) for 30 min at 37 °C. qPCR was performed using an Applied Biosystems StepOnePlus^{TM} System and Fast SYBR Green PCR Master Mix (Applied Biosystems). *RpL32* was used as a calibrator and verified in our experiments as a reliable standard unaffected by *Pe* treatment[6]. Transcript levels relative to *rpl32* were calculated using the $2^{-\Delta CT}$ method and were normalized to mock treated wild type levels for display purposes.

Microbial exposure
Natural infections were performed by exposing adult flies to *Pseudomonas entomophila (Pe) ad libitum*. Exposure was achieved by transferring adults to standard fly food vials covered with Whatman filter paper supplemented with 0.2 mL of *Pe* suspension. *Pe* bacterial culture was concentrated by centrifugation and delicately resuspended in 5% sucrose. OD_{600} was measured and the concentrated *Pe* was subsequently diluted to the designated experimental OD. Mock-infected flies were placed on fly food covered with Whatman filter paper supplemented with 0.2 mL 5% sucrose. Flies were shifted to and maintained at 29°C throughout the course of the experiment following mock or *Pe* treatment. Exposure to mock or *Pe* containing food was for 24 h then flies were subsequently returned to standard food media and survival was scored. For experiments involving low titer infection (OD_{600}=5 or 10) where wild type lethality is very low, it is necessary to independently validate the pathogenicity of each experimentally prepared pellet that will undergo dilution for the low titer studies. This is achieved by running a series of wild type diagnostic infections for each experiment. Diagnostic infections consists of three vials containing 20 flies/vial exposed to OD_{600}=20 and run in parallel to low titer infections derived from the same pellet. Only pellets showing the anticipated LD50 at OD_{600}=20 were subsequently analyzed in the low titer condition. All low titer infection data presented corresponds to pellets for which the LD50 was empirically validated in this manner. Diagnostic vials were not used for high titer infections (OD_{600}=100) where the LD50 is readily evident. Systemic infections for Relish immunostaining were performed by pricking the larval cuticle with a thin needle dipped in a bacterial suspension of *Pe* or *Erwinia carotovora carotovora 15 (E.cc15)* and fixed 1 h post exposure.

Survival analysis
0-2 day old progeny of the appropriate genotype were collected. 20 females and 2 males were placed in a single fresh food vial and aged for three days before exposure to *Pe or E.cc15*. Flies were transferred onto fresh food after 24 h and every 2 days subsequently, for the duration of the experiment. The number of surviving female flies was scored.

Mutant rescue assay
To minimize environmental differences between the genotypes used in the mutant rescue experiments a cohabitation protocol was used. Parental crosses of the following genotypes were established in individual vials and allowed to mate for 3 days: 1) w^{1118} x w^{1118}, 2) w^{1118}; ima^{Δ}/CyO, actin-GFP; +/+ x w^{1118}; ima^{Δ}/CyO, actin-GFP; daGal4/TM6C, 3) w^{1118}; ima^{Δ}/CyO, actin-GFP; UAS-rescue/TM6C x w^{1118}; ima^{Δ}/CyO, actin-GFP; daGal4/TM6C. Males were discarded and mated females from all three crosses were then pooled into a single bottle where they were allowed to lay eggs for 2-3 days. F1 progeny derived from this egg lay developed in the same environment together and then for an additional 2 days of adulthood following eclosion. Non-balancer flies were next sorted by eye color to genetically follow the presence of mini w+ transgenes (i.e. white eye, w^{1118} / w^{1118}; yellow eye, w^{1118} / w^{1118}; ima^{Δ}/ ima^{Δ} ; daGal4/+; dark orange eye, w^{1118} / w^{1118}; ima^{Δ}/ ima^{Δ} ; daGal4/UAS-rescue), then transferred to individual vials to facilitate scoring survival, aged for 3 days and challenged with *Pe*.

Statistical analysis
All statistical analysis was performed using Prism GraphPad Software. The sample size (n) is defined as the total number of independent observations per treatment group. All data show the mean of at least three independent experiments and the error bars used denote standard error (SE). The specific statistical test used for each experiment is indicated in the figure legends. Statistical significance is shown as follows; N.S.; * $P < 0.05$; ** $P < 0.01$; *** $P < 0.001$; **** $P < 0.0001$

Bioinformatics
Direct comparison of *Drosophila* and human full-length proteins was performed using blastp multiple alignment, available online through NCBI (https://blast.ncbi.nlm.nih.gov/Blast.cgi). All sequences for LysMD3/4, LysMD3, and LysMD4 proteins were obtained from NCBI Protein Database using a query search for “LysM domain.” From this LysMD master list, we removed all proteins that did not fit for our analysis, which included many partial protein sequences and mislabeled non-LysM domain-containing proteins. We also removed isoforms of the same protein. Sequences were then aligned using MUSCLE alignment available in MEGAX (https://doi.org/10.1093/molbev/msz312). Aligned sequences were trimmed by hand, removing any amino acids that did not have a conservation score of at least 1. Phylogenetic analysis was performed using the BEAUTI/BEAST software package, version 1.10.4[7]. Bayesian analysis was performed using the WAG, Gamma Site Substitution Model with a gamma category count of 4 and the Yule Model for speciation. Priors and Operators were tuned following recommendations from BEAST software output. Chain lengths for the complete phylogeny (pan-Metazoa) and the limited phylogeny (Vertebrata, Insect, and Cnidaria) were 5,000,000 and 100,000, respectively. Maximum clade credibility trees were determined using a burnin of 10%. Fasta files of sequence

alignments and XML files of parameters for BEAST analysis are available in Supplementary Data.

Microscopy
Generation of whole mount samples for immunostaining was performed using standard methodology. Briefly, tissues were fixed 1x PBS and 4% EM-grade formaldehyde (Polysciences) for 30 min (larval tissue) or overnight (adult tissue), washed in 1x PBS, 0.1% Triton X-100 (PBST) for a minimum of 2 hours, and incubated in primary antisera overnight. Samples for Relish immunostaining were permeabilized with 1% Triton for 15 min then washed and blocked with PBST containing 0.1% BSA and 6% normal goat serum. Samples were washed and incubated in secondary antibodies for 3 hours, washed again in PBST, and mounted in Vectashield+DAPI. All steps were completed at 4°C. *Antisera*: mouse anti-p120 Golgi (1:100 Calbiochem); rabbit anti-Relish 130-10080 (1:300, RayBiotech); chicken anti-GFP (1:10000, Abcam); mouse anti-Golgin84 (1:100, DSHB clone 12-1); guinea pig anti-GMAP (1:2000, DSHB); mouse anti-Hrs (1:100, DSHB clone 27-4); Alexa Fluor conjugated secondary antibodies (1:2000, Molecular Probes).

Whole mount samples were analyzed on a Leica DM5000 compound or Leica TCS SP5 confocal microscope. Images were processed for brightness and contrast in Photoshop CS (Adobe). Fluorescence signal quantitation was performed using ImageJ and colocoalization analysis was performed using the JACoP plugin for ImageJ.

Immuno-electron microscopy was performed by the Center for Cellular Imaging at the Washington University in St Louis. w^{1118} and *ima::GFP* salivary glands were processed for immuno-electron microscopy by fixing in 4% formaldehyde in PBS. For immunoperoxidase – 3,3’-diaminobenzidine (DAB) staining, specimens were washed 3X in PBS 10 min. ea., blocked using 1% NaBh4 in PBS 15 min., rinsed again in PBS 3X 10 min ea., then incubated with primary antibody to GFP overnight at 4 °C. Two antibodies were used 1) Abcam polyclonal rabbit anti-GFP (ab6556) diluted 1:2500 in PBS and 2) Abcam biotinylated polyclonal goat anti-GFP (ab6658) diluted 1:1000 in PBS. Negative controls excluded primary antibodies. After overnight incubation, samples labeled with rabbit anti-GFP, ab 6556, were washed 3X in PBS, then incubated with biotinylated donkey anti-rabbit (Jackson ImmunoResearch Laboratories, West Grove, PA) diluted 1:1000 in PBS for 2 h. with gentle agitation. The remaining steps were the same for both primary antibodies. Samples were washed in PBS 3X 10 min ea. and incubated in ABC solution (ABC Vectorlabs Vecastain Elite kit, (Burlingame, Ca) for 45 min with agitation. Samples were washed in PBS 3X 10 min ea., washed in 0.1 M sodium acetate buffer 2X 5 min ea., then DAB stained with Nickel Dab solution (20 mg beta d+ glucose, 4 mg ammonium chloride, 5ml of 5% nickel ammonium sulfate in 0.2 M sodium acetate buffer, 4 ml H2O, 1 ml 5mg/ml DAB solution 1 mg glucose oxidase), for 2-5 min. Once light brown DAB precipitate formed samples were rinsed 3X 5 min ea. in 0.1 M sodium acetate buffer to stop the

reaction, washed with PBS 3X 5 min ea., and fixed with 2.5% glutaraldehyde, 2% paraformaldehyde in PBS buffer. Samples were post fixed with 1% OsO4, 1.5% potassium ferrocyanide in 0.15 M cacodylate buffer for 1 h, washed 3X 10 min ea., and *en bloc* stained with 2% uranyl acetate for 1 h in the dark. They were washed in milliQ water 2X 10 min ea., dehydrated in a graded acetone series (50, 70, 90, 100% X2) 10 min ea., infiltrated in LX110 with microwave assistance(350 W, 5 min with vacuum) starting with 50% resin/acetone, then 2 changes of 100% resin. Samples were embedded in molds and polymerized at 60 °C for 48 h. Sections 70 nm thick were made, picked up on 100 hex mesh copper grids (Electron microscopy Sciences, PA) and viewed using a JEOL 1400 plus transmission electron microscope equipped with an AMT (AMT Imaging, Woburn, MA) CCD camera system. Golgi structures, which in the *Drosophila* salivary glands appear as clusters of vesicles and tubules, were identified as previously described[8-10].

Extended experimental procedures references

1. F. Port, H.-M. Chen, T. Lee, S. Bullock, Optimized CRISPR/Cas tools for efficient germline and somatic genome engineering in *Drosophila. Proc. Natl. Acad. Sci. U.S.A.* **111**, 2967-2976 (2014).

2. F. Port, N. Muschalik, S. Bullock, Systematic Evaluation of *Drosophila* CRISPR Tools Reveals Safe and Robust Alternatives to Autonomous Gene Drives in Basic Research. *G3* **20**, 149-14502 (2015).

3. U. Berstche, A.A. Gust, Peptidoglycan Isolation and Binding Studies with LysM-Type Pattern Recognition Receptors. *Methods Mol Biol.* **1578**, 1-12 (2017).

4. L. Abas, C. Luschnig, Maximum yields of microsomal-type membranes from small amounts of plant material without requiring ultracentrifugation. *Anal Biochem.* **401**, 217-227 (2010).

5. B. Liu, Lysin Motif–Containing Proteins LYP4 and LYP6 Play Dual Roles in Peptidoglycan and Chitin Perception in Rice Innate Immunity. *Plant Cell* **24**, 3406-3419 (2014).

6. K. Beebe, D. Park, P.H. Taghert, C.A. Micchelli, The Drosophila Prosceretory Transcription factor dimmed Is Dynamically Regulated in Adult Enteroendocrine Cells and Protects Against Gram-Negative Infection. *G3* **5**, 1517-1524 (2015).

7. A. Drummond, M.A. Suchard, D. Xie, A. Rambaut, Bayesian Phylogenetics with BEAUti and the BEAST 1.7 *Mol. Biol. Evol.* 29(8), 1969-1973 (2012).

8. V. Kondylis, C. Rabouille, The Golgi apparatus: Lessons from Drosophila. *FEBS Lett.* **583**, 3827-3838 (2009).

9. S. Wang, H. Meyer, A. Ochoa-Espinosa, U. Buchwald, S. Onel, B. Altenhein, J. Heinisch, M. Affolter and A. Paululat, GBF1 (Gartenzwerg)-dependent secretion is required for Drosophila tubulogenesis. *J Cell Sci.* **125**, 461-471 (2011).

10. R. Fox, D. Andrew, Changes in organelle position and epithelial architecture associated with loss of CrebA. *Biol Open.* **4**, 317-330 (2015).
